# Transcriptomic Plasticity in the Small Hive Beetle (*Aethina tumida*) Under Heat Stress

**DOI:** 10.3390/insects16080868

**Published:** 2025-08-21

**Authors:** Junfeng Liu, Yuxiang Wang, Yuzhu He, Keyue Jin, Xiaojuan Wan, Danwei Chen, Tailin Zhong, Xujiang He, Guoyun Wu

**Affiliations:** 1 College of Animal Science and Technology, Jiangxi Agricultural University, Nanchang 330029, China; liujunfeng@jxau.edu.cn (J.L.); 18456662422@163.com (Y.W.); heyuzhu@stu.jxau.edu.cn (Y.H.); keyuer42@163.com (K.J.); xujianghe@jxau.edu.cn (X.H.); 2 School of Basic Medical Sciences, Jiangxi Medical College, Nanchang University, Nanchang 330047, China; wanxiaojuan8373@ncu.edu.cn; 3 College of Urban Construction, Zhejiang Shuren University, Shaoxing 312028, China; 600987@zjsru.edu.cn

**Keywords:** *Aethina tumida*, heat resistance, survival ability, gene expression, qPCR

## Abstract

Global warming is increasing in severity, affecting insects across various biological species. This study investigated the heat resistance ability of the small hive beetle (*Aethina tumida*) by studying gene expression under heat stress and showed that *A. tumida* exhibits strong heat resistance and transcriptomic plasticity under heat stress. RNA-seq analysis identified 547, 1127, and 866 differentially expressed genes (DEGs) at 38 °C, 42 °C, and 46 °C, respectively, compared to 25 °C. Among them, 16, 25, and 5 heat shock protein (*HSP*) genes were differentially expressed under the three heat stress conditions. Specifically, one *HSP70* gene (Loc109602670) was consistently upregulated across all temperatures. Furthermore, the lysosome-related pathway was the top enriched pathway under heat treatments, with key genes such as *lysosomal aspartic protease-like*, *cathepsin L1-like*, and *lipase 3-like* significantly upregulated. Overall, these findings suggest that *A. tumida* exhibits transcriptomic plasticity under sublethal heat stress, and key *HSP* genes with genes from lysosome pathways are likely to contribute to heat resistance. This study provides novel insights into the molecular basis of thermotolerance in *A. tumida*, contributing to our understanding of how this invasive pest adapts to high-temperature environments.

## Simple Summary

The small hive beetle (*Aethina tumida*) represents a significant threat to apiculture worldwide. This study explores the molecular mechanisms underlying heat tolerance in the invasive pest *A. tumida*. Using transcriptome analysis, we found that this species exhibits strong thermotolerance and remarkable gene expression plasticity under heat stress. In particular, our findings suggest that heat shock proteins and lysosome-related pathways may play important roles in the beetle’s stress response. These findings provide new insights into how *A. tumida* adapts to rising temperatures, offering a molecular foundation for understanding insect resilience in the context of climate change.

## 1. Introduction

Due to the global warming, unexpected high temperatures have been frequent in these years. Insects are generally small and are sensitive to heat stress. A recent study used 102 insect species for meta-analyses and revealed that insects exhibit a limited capacity to acclimate their thermal tolerance to elevated temperatures, which renders them more susceptible to the impacts of global warming than previously estimated [1]. They can be influenced by high temperature in terms of their development, phenotype, reproduction, survival, and population dynamic [2,3,4]. For example, In *Coccophagus japonicus*, heat stress shortened adult lifespan, reduced egg production, and altered developmental duration [3]. Similarly, in *Spodoptera frugiperda*, heat stress decreased survival and reduced pupal weight, potentially affecting long-term population dynamics [4]. A current survey showed that the diversity of pollinating bees has decreased rapidly due to high temperatures [5]. Therefore, to understand heat resistance in some special insect species is increasingly important to help other species that are highly sensitive to high temperatures.

A few insect species have good heat resistance. For example, the Saharan silver ant (*Cataglyphis bombycina*) has developed a unique thermoregulatory adaptation through specialized reflective hairs that minimize solar radiation absorption, enabling activity during peak desert heat with an air temperature over 50 °C [6,7]. The male desert locust (*Schistocerca gregaria*) extends legs to lift its body off the hot ground and positioned itself parallel to the sun’s rays, minimizing radiative heating [8]. Some desert beetles (such as *Tenebrionidae* and *Chrysomelidae*) are among the most successful animals in adapting to the extremely high-temperature habitats of the desert [9].

The small hive beetle, *Aethina tumida*, is a beetle species belonging to the family *Nitidulidae*, native to sub-Saharan Africa. It is also well known as a significant pest of honeybee colonies and poses a major threat to apiculture worldwide [10]. Differing from the above Saharan silver ants and desert beetles that are mainly adapted to the dry and extreme high-temperature desert environment, this small hive beetle is well-adapted to various environmental habitats and is widespread across Africa, America, Europe, Australia, and Asia [11]. It can cause severe damage to honeybee colonies by feeding on honey, pollen, and brood, leading to the fermentation of hive products and ultimately colony collapse, resulting in significant economic losses for beekeepers due to reduced honey production and the abandonment of hives by bees [10]. Understanding the heat tolerance of the small hive beetle is important for protecting honeybee health. As a heat-resistant invasive pest, it poses a growing threat to colonies, especially under climate change. Studying its thermal tolerance can reveal how it survives in extreme conditions and help assess potential health risks to bees.

Noor-ul-Ane and Jung (2020) showed that this small hive beetle has good heat resistance and predicted that the high developmental threshold temperatures for this beetle would be 40.4 to 46.5 °C [12], though high temperature could affect the development of its larvae [13]. Therefore, although this beetle does not appear to possess specialized strategies for heat resistance, it still exhibits notable thermal tolerance. Investigating the underlying molecular mechanisms could help us to understand the heat resistance ability of this pest and offer suggestions for its management and control. It may also be important for heat-vulnerable insects, rather than for species adapted to extreme heat stress, such as the Saharan silver ant.

The heat resistance molecular mechanisms of small insects are still not well understood. King and MacRae (2015) reviewed the key role of heat-shock protein (*Hsp*) genes in insect heat resistance, including *Hsp60*, *Hsp70*, and *Hsp90* and some small heat shock proteins (*sHsps*) [14]. *Hsps* facilitate numerous essential molecular processes in insects, such as protein folding, localization, and degradation, helping maintain protein homeostasis under thermal stress [14]. Heat stress induces a high expression of *Hsps* in insects, which bind misfolded proteins and aid refolding. For example, heat stress induces the upregulation of *Hsp90* and *Hsp70* in apple maggot fly (*Rhagoletis pomonella*), enhancing its thermal tolerance [15]. Similarly, *sHsp* genes such as *sHsp19.9* and *sHsp20.4* from silkworm (*Bombyx mori*) are highly expressed in the larval fat body, testis, and ovary under thermal stress [16]. *Hsp40*, *Hsp20*, *Hsp70*, *Hsp90*, and mitochondrial *Hsp60* play an important role in both heat and cold tolerance in two leafminers (*Liriomyza sativa* and *Liriomyza huidobrensis*) [17].

In response to heat stress, insects rely not only on heat shock proteins but also on a broad array of other molecular mechanisms. Genes or proteins involved in the oxidative stress response are increasingly recognized for their roles in insect thermotolerance. For instance, superoxide dismutase, catalase and glutathione S-transferase enzymes contribute to cellular protection by mitigating heat-induced oxidative damage [18]. Moreover, enzymes or related genes involved in energy metabolism and chitin synthase are also involved into insect heat resistance. For example, the potato aphid raises the concentrations of enzymes for ATP generation and circular protein biosynthesis during heat stress [19]. Therefore, the *Hsp* family genes and other related genes are possibly regulated in a coordinated manner, forming a complex and integrated network that underlies the insect’s adaptive response to thermal stress. However, the molecular mechanisms underlying insect heat resistance remain incompletely understood and may involve more interacting pathways than currently documented.

Based on previous evidence that *HSPs* act as primary molecular chaperones under thermal stress and that other genes contribute to cellular protection, we hypothesized that some key genes enriched in specific pathways, together with *HSPs*, may constitute the core responders to heat stress in *A. tumida*. Here, we used *A. tumida* as a model to investigate the molecular basis of insect thermotolerance. We reared the small hive beetle under 25 °C, 38 °C, 42 °C, and 46 °C, and their survival ability was evaluated. We also sequenced RNA samples from insects in the above conditions by RNA-seq and qPCR to explore *A. tumida* gene expression profiles related to thermal tolerance, which may contribute significantly to enriching the theoretical research on insect heat tolerance mechanisms.

## 2. Materials and Methods

### 2.1. Insects

Thousands of small hive beetles were provided by eight honeybee apiaries in Haikou city, China, and were reared in 10 plastic boxes with a diet consisting of 40% honey, 40% rapeseed pollen, and 20% yeast extract powder (purity 90%, Beijing Hongrun Baoshun Technology Co., Ltd., Beijing, China) under constant room temperature of 30 °C in an incubator (YT-SPX-150BE, Shandong Yuntang Intelligent Technology Co., Ltd., Weifang, China). Totally, 560 newly emerged healthy beetles with similar body size were randomly harvested from the 10 plastic boxes on the same day. For the survival test, 400 beetles were divided into four groups and placed at 25 °C, 38 °C, 42 °C, and 46 °C. Each group had 100 beetles. For the RNA-Seq and qPCR analyses, 160 beetles were divided into four groups as above.

### 2.2. Survival Test on the Small Hive Beetle

The small hive beetles of each group were transferred into a stainless-steel container (diameter: 10 cm), which was subsequently placed in a water bath (SHJ-4AB, Changzhou Jintan Liangyou Instrument Co., Ltd., Changzhou, China) for heat stress treatment at 25 °C, 38 °C, 42 °C, and 46 °C. The beetles were subjected to heat stress without access to food or water, as the presence of food or water could potentially buffer ambient temperature fluctuations and compromise the stability of the heat stress treatment. We inspected and counted the dead beetles every 0.5 h, and LD_50_ and LD_95_ were recorded.

For RNA-Seq and qPCR, the remaining 160 beetles were divided and treated under four temperatures as above but only heated for 0.5 h, an exposure we deemed optimal for assessing heat resistance while minimizing the potential effects of starvation on gene expression. The beetles were then harvested for RNA-Seq and qPCR. Each sample contained four beetles for RNA-Seq or qPCR, and each treatment had three biological replicates. The beetles in a sample were placed into a 1.5 mL microcentrifuge tube and directly immersed in liquid nitrogen for cryopreservation and storage. In total, 12 samples for RNA-Seq and another 12 samples for qPCR were analyzed.

### 2.3. RNA Extraction and Sequencing

The total RNA of each sample was extracted using Invitrogen RNA extraction kits (Thermo Fisher Scientific, Waltham, MA, USA) following the manufacturer’s instructions. RNA concentration and purity were measured using an Agilent 2100 Bioanalyzer (Agilent Technologies, Santa Clara, CA, USA).

In total, 1 µg of each RNA sample with high quality (RIN > 7) was used for library preparation for RNA-Seq. mRNA was enriched from total RNA using oligo(dT) magnetic beads and subsequently fragmented into short fragments. First-strand cDNA was synthesized using random hexamer primers and reverse transcriptase, followed by second-strand cDNA synthesis. The resulting cDNA products were purified using the AMPure XP magnetic bead system and then end-repaired, A-tailed, and ligated to sequencing adapters. Size selection of DNA fragments was conducted using AMPure XP beads. After purification and PCR amplification, the final cDNA libraries were quantified and validated for size distribution using the bioanalyzer. The qualified libraries were then sequenced on an Illumina NovaSeq 6000 platform (Illumina, San Diego, CA, USA) using the paired-end 150 bp (PE150) strategy to generate high-throughput sequencing data.

### 2.4. Data Processing and Expression Quantification

To ensure the accuracy and reliability of the downstream analyses, the raw sequencing reads were first subjected to a comprehensive quality control process using fastp (version 0.23.2). This procedure included the removal of adapter sequences and reads originating from adapter self-ligation events that failed to capture the target fragments. Bases with Phred quality scores below 20 at both ends of the reads were trimmed, and reads containing bases with a quality score below 10 after trimming were discarded. Additionally, reads containing ambiguous nucleotides (“N”) or those shorter than 30 bp following quality filtering were excluded. After quality control, clean reads were retained for further analysis and quality reassessment.

### 2.5. Gene Expression Analysis

Low-quality reads were filtered out, and only those with a sequencing error rate below 1% (Q20 > 98%) were retained. The clean reads were mapped to the genome of *A. tumida* (icAetTumi1.1). Transcript and gene expression levels were quantified using Cufflinks, employing the Cuffquant and Cuffnorm modules [20]. Expression abundance was calculated based on the alignment of reads to annotated gene features and normalized as fragments per kilobase of exon per million mapped fragments (FPKM), enabling accurate cross-sample comparisons of the expression profiles. Gene expression after the four treatments was compared using edgeR [21], and DEGs were identified based on *p* value < 0.01 and log2 fold change (Log2FC) ≥ 1.5. Moreover, to assess the overall similarity and variation among the transcriptomic samples, PCA analysis was performed based on the expression profiles of all detected genes from the 12 samples using PCA analysis tools on the platform BMKCloud (www.biocloud.net, accessed on 15 November 2021).

### 2.6. Enrichment Analysis of GO and KEGG

All DEGs from three comparisons (38 °C vs. 25 °C, 42 °C vs. 25 °C, and 46 °C vs. 25 °C) were aligned against various protein and nucleotide sequence databases using BLASTX+2.12.0, including the NCBI non-redundant protein (Nr) database, the Swiss-Prot protein database, and the non-redundant nucleotide (Nt) database, with a cut-off E-value of 10^−5^. GO enrichment analysis was performed by mapping the DEGs to Gene Ontology (GO) terms, and significantly enriched GO terms were identified using a hypergeometric test (*p* < 0.05) [22].

Subsequently, the DEGs from each of the above comparisons were mapped to the Kyoto Encyclopedia of Genes and Genomes (KEGG) protein database (http://www.genome.jp/kegg/kegg1.html, accessed on 15 November 2021) using BLAST+2.12.0 (E-value < 10^−5^). KEGG pathway enrichment was assessed using the KOBAS 2.0 software [23], and statistical significance was determined by a hypergeometric test with a Q-value threshold of <0.05.

### 2.7. qRT-PCR Verification of Eight Genes

Total RNA extracted from the 12 samples was normalized prior to reverse transcription, similar to our previous study [24]. cDNA was synthesized using MLV reverse transcriptase (Takara, Osaka, Japan) in accordance with the manufacturer’s guidelines. The *β*-actin gene was employed as an internal reference gene. Eight genes were randomly selected from the RNA-Seq data for qRT-PCR validation. The primers for these genes were designed using Primer 5.0 software and are presented in Table S1. qPCR was performed using the ABI 7500 Real-Time PCR System (Applied Biosystems, Rockville, MD, USA) with a 20 μL SYBR Green reaction mixture consisting of SYBR Green master mix 10 μL, forward primer 0.4 μL, reverse primer 0.4 μL, cDNA template 1 μL, and nuclease-free water 8.2 μL. The qPCR cycling conditions were as follows: 94 °C for 2 min, 40 cycles, followed by 94 °C for 15 s, 60 °C for 30 s, and 72 °C for 30 s. The QuantStudio™ 5 System Real-Time-PCR Instrument (Thermo Fisher Scientific Inc., Waltham, MA, USA) was used for qPCR data analysis. Melt curve analysis was conducted for each sample to confirm the specificity of the PCR amplification products. For each gene, three independent biological replicates were analyzed, each with five technical replicates. To minimize inter-plate variability, both reference and target genes from the same sample were assayed on the same qPCR plate. The cycle threshold (Ct) value for each biological replicate was determined by averaging the values from three technical replicates. The relative expression level of each gene from the four treatments was determined using the 2^−ΔΔCt^ method.

### 2.8. Data Analysis

**Figure 1:**
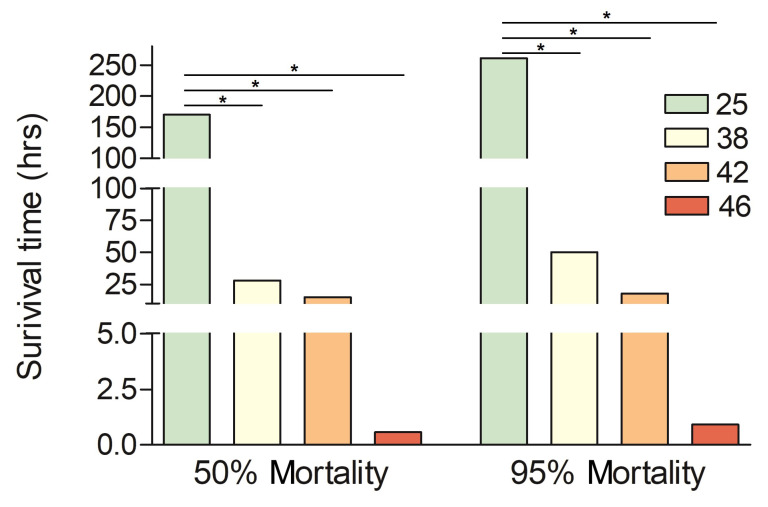
Survival time of *A. tumida* at LD50 and LD95 under 25 °C, 38 °C, 42 °C, and 46 °C. “*” represents a significant difference between the heat-stressed group and the control group (*p* < 0.05, Kruskal–Wallis test).

The survival data of the small hive beetle from the four treatments (Figure 1) were compared using Kruskal–Wallis test in Statview 5.0 (SAS Institute Inc., Cary, NC, USA), and a *p* value < 0.05 was considered as indicating a significant difference. The relative expression levels of the 9 genes in the qRT-PCR experiment were calculated using the 2^−ΔΔCt^ format and then analyzed using the ANOVA test followed by Fisher’s LSD test in Statview, and a *p* value < 0.05 was considered as indicating a significant difference.

## 3. Results

### 3.1. The Survival Ability of Small Hive Beetles Under Heat Stress

The small hive beetles could survival several days under room temperature (25 °C, 50% mortality time: 170 h, 95% mortality time: 261 h). Increasing the environmental temperature raised the death ratio of the beetles, with values of 28 h (50% mortality time) and 50 h (95% mortality time) at 38 °C, 15 h (50% mortality time) and 18 h (95% mortality time) at 42 °C, and 0.58 h (50% mortality time) and 0.92 h (95% mortality time) at 46 °C (Figure 1). These findings show that the small hive beetle has good survival ability under heat stress.

### 3.2. Quality of the RNA Sequencing (RNA-Seq) Data

Transcriptome analysis was conducted with 12 samples including 25 °C-, 38 °C-, 42 °C-, and 46 °C-treated larval groups (each group had three replicates). In total, 255.35 GB of clean data were obtained. The clean data of each sample were 21.28 GB, and the percentage of Q30 bases was over 92% (Table S2). These results revealed that the RNA-Seq quality of all samples was high, and the data were reliable. Pearson’s correlation coefficient of all biological replicates in each group was above 0.9 (Figure S1), indicating that there was acceptable sequencing quality and repeatability among the biological replicates of each group.

**Figure 2:**
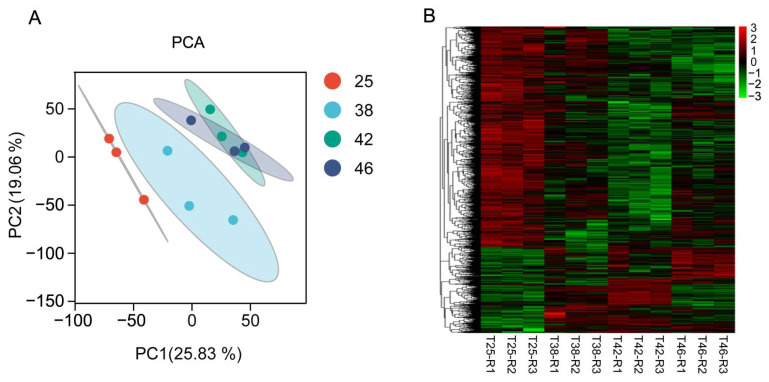
Comparison of gene expression patterns among the 12 RNA-Seq samples. (**A**) Principal component analysis (PCA) of the four treatments; (**B**) gene expression patterns of the 12 samples. The color scale is the log10 (FPKM + 1) value of each gene.

The results of principal component analysis (PCA) showed a clear separation between the three heat-stressed groups (38 °C, 42 °C, and 46 °C) and the control group (25 °C), indicating distinct transcriptomic patterns induced by heat stress in *A. tumida* (Figure 2A). Specifically, the 46 and 42 °C groups clustered closely together, while samples from the 38 °C group were distinctly separated (Figure 2A). The control and heat-stressed groups also showed different expression patterns of all genes (Figure 2B). These findings revealed that all high-temperature groups had a similar gene expression pattern, which considerably differed from that of the control.

### 3.3. Significantly Differentially Expressed Genes (DEGs)

**Figure 3:**
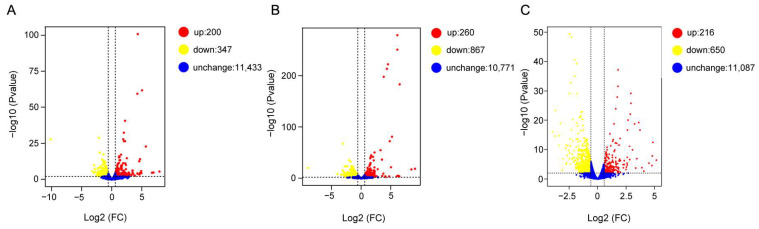
Volcano diagrams of DEGs from the 25 °C vs. 38 °C, 25 °C vs. 42 °C, and 25 °C vs. 46 °C comparisons. (**A**) DEGs from the 25 °C vs. 38 °C comparison; (**B**) DEGs from the 25 °C vs. 42 °C comparison; (**C**) DEGs from the 25 °C vs. 46 °C comparison. The red dots indicate upregulated DEGs in the heat-stressed groups compared to the control, the yellow dots indicate downregulated DEGs in the heat-stressed groups, and the blue ones indicate non-differentially expressed genes. The DEGs were identified based on *p* value < 0.01 and Log2FC < 1.5.

We identified 547, 1127, and 866 DEGs when comparing samples at 38 °C vs. 25 °C, 42 °C vs. 25 °C, and 46 °C vs. 25 °C, respectively (Figure 3, for details see Tables S3–S5).

### 3.4. Gene Ontology (GO) and Kyoto Encyclopedia of Genes and Genomes (KEGG) Pathway Enrichment Analysis

The Gene Ontology (GO) enrichment results for DEGs under different heat stress conditions relative to the control temperature (25 °C) revealed distinct biological responses across temperature gradients. At 38 °C (Figure S2A), the DEGs were significantly enriched in GO terms associated with metabolic process, cell and extracellular parts, protein folding, and catalytic activities. At 42 °C (Figure S2B), DEG enrichment expanded beyond earlier responses, with notably increased representation in categories related to behavior, detoxification, antioxidant activity, and rhythmic processing. At 46 °C (Figure S2C), GO terms related to immune system processes, cell junctions, oxidative stress response, and electron carrier activity showed increased enrichment compared to the 38 °C and 42 °C treatments.

**Figure 4:**
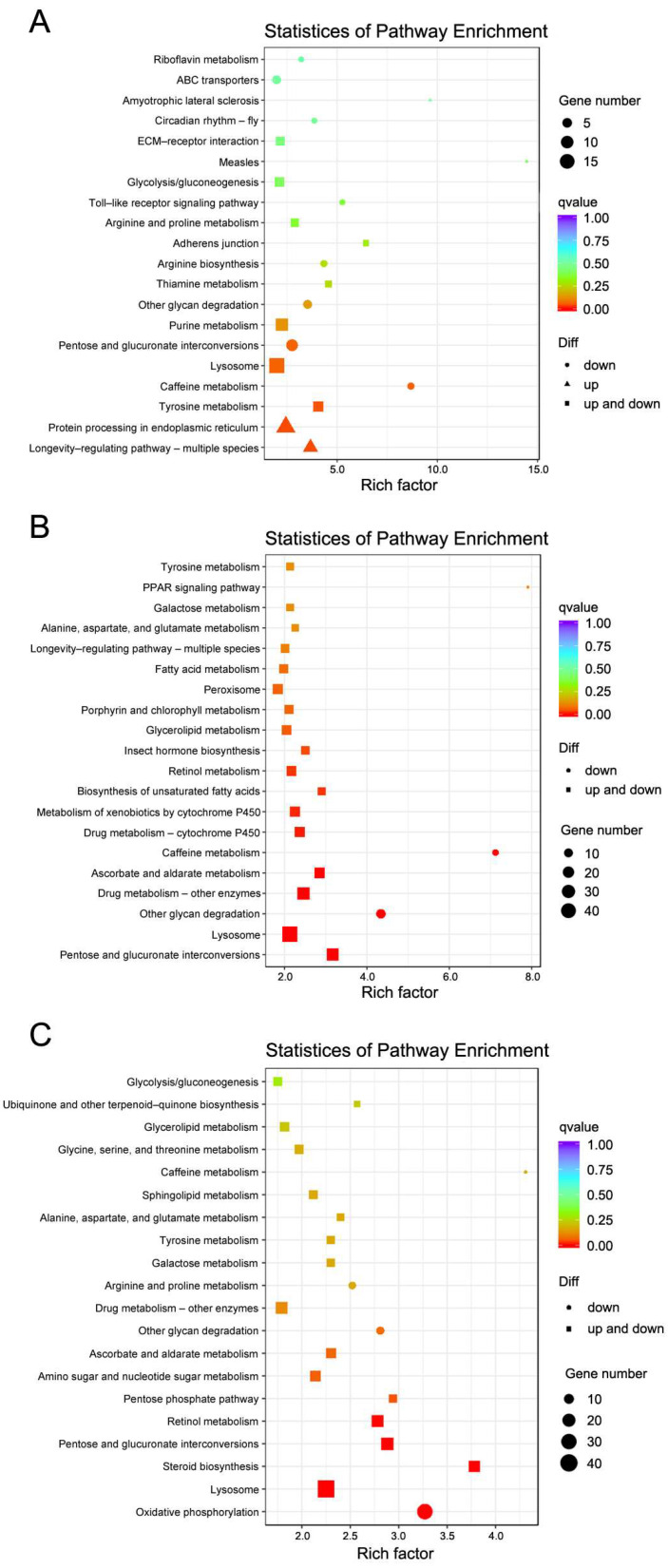
Top 20 KEGG pathways from the heat-stressed and control groups. (**A**) Top 20 KEGG pathways in the 25 °C vs. 38 °C comparison; (**B**) top 20 KEGG pathways in the 25 °C vs. 42 °C comparison; (**C**) top 20 KEGG pathways in the 25 °C vs. 46 °C comparison. Circles, triangles, and squares represent downregulated, upregulated, and both up- and downregulated DEGs in heat-stressed groups, respectively. The color scale represents the q values, and the size of the shapes represents the number of DEGs.

The KEGG results clearly showed that the lysosome pathway was the top one pathway in all three comparisons, and its proportion increased with the temperature, corresponding to 10.34%, 11.08%, and 11.71% in the 38 °C vs. 25 °C, 42 °C vs. 25 °C, and 46 °C vs. 25 °C comparisons, respectively (Figure 4). The following top pathways in the 38 °C vs. 25 °C comparison were protein processing in endoplasmic reticulum, purine metabolism, pentose and glucuronate interconversions, and longevity-regulating pathway. In the 42 °C vs. 25 °C and 46 °C vs. 25 °C comparisons (Figure 4A), the top pathways were slightly different, with drug metabolism-other enzymes, pentose and glucuronate interconversions, ascorbate and aldarate metabolism, and metabolism of xenobiotics by cytochrome P450 in the 42 °C vs. 25 °C comparison (Figure 4B), and oxidative phosphorylation, pentose and glucuronate interconversions, retinol metabolism, and drug metabolism-other enzymes in the 46 °C vs. 25 °C comparison (Figure 4C). Consequently, these results indicate the importance of the lysosome pathway in heat stress resistance in small hive beetles.

### 3.5. Expression of Key Heat Shock Family Genes

**Figure 5:**
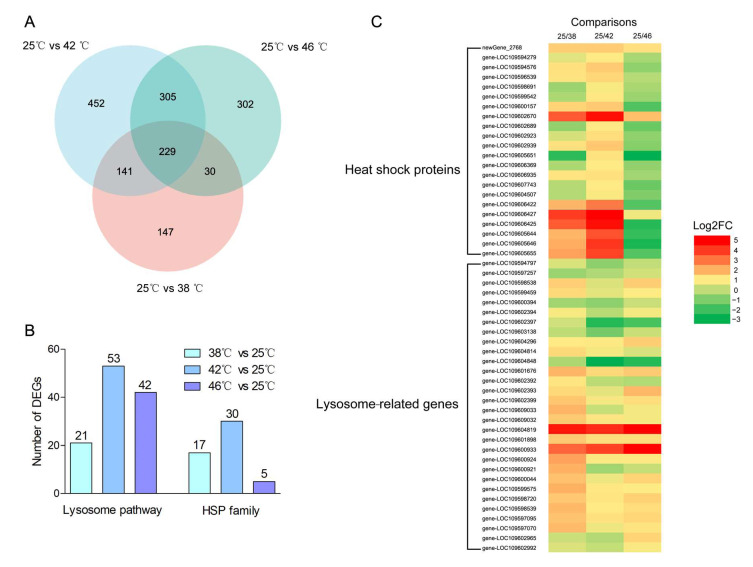
(**A**): Venn diagram of the DEGs from the three comparisons (25 °C vs. 38 °C, 25 °C vs. 42 °C, and 25 °C vs. 46 °C); (**B**) number of DEGs enriched in the lysosome pathway and HSP family between the heat-stressed and the control groups; (**C**) heat map of key DEGs enriched in the lysosome pathway and HSP family. The red bars represent upregulated DEGs in the heat-stressed groups, and the green bars represent downregulated DEGs in the heat-stressed groups.

Heat stress induced many DEGs from the heat shock gene family, with 16, 25, and 4 heat shock protein (*HSP*) genes in the 38 °C vs. 25 °C, 42 °C vs. 25 °C, and 46 °C vs. 25 °C comparisons, respectively (Figure 5B). These DEGs were primarily from the *HSP70*, *HSP20*, and *HSP90* families. Interestingly, the number of significantly differentially expressed *HSPs* increased in the 42 °C vs. 25 °C comparison compared to the 38 °C vs. 25 °C comparison, while this number decreased in the 46 °C vs. 25 °C comparison (Figure 5C). There were 299 overlapping DEGs among the three comparisons (Figure 5A). One *HSP70* gene (Loc109602670) was the unique *HSP* family gene significantly differentially upregulated in all three comparisons.

### 3.6. Key DEGs Enriched in the Lysosome Pathways

We identified 21, 53, and 42 DEGs that were enriched in the lysosome pathway from the 38 °C vs. 25 °C, 42 °C vs. 25 °C, and 46 °C vs. 25 °C comparisons, respectively (Figure 5B). Notably, 10 key DEGs, including the *partic protease-like*, *Cathepsin L1-like*, and *Lipase 3-like* genes, which play an important role in the lysosome pathway, overlapped in all three comparisons (Figure 5C).

### 3.7. RT-qPCR Verification

**Figure 6:**
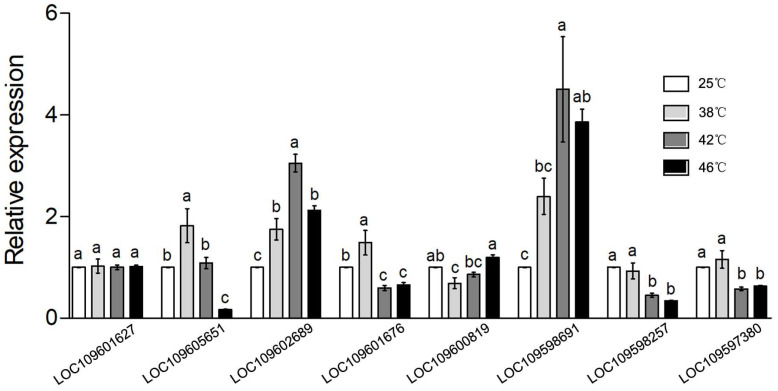
The qPCR of eight genes from the 25 °C, 38 °C, 42 °C, and 46 °C groups. Same letters on the bars represent no significant difference (*p* > 0.05, ANOVA test), different letters represent a significant difference (*p* < 0.05).

The results of qPCR showed that the expression of six of eight DEGs from RNA-Seq results was consistent with the results of RNA-seq, including the expression of *heat shock 70 kDa protein cognate 5-like* (LOC109602689), *lysosomal aspartic protease-like* (LOC109601676), *cytochrome b ascorbate-dependent protein 3 isoform X1* (LOC109600819), *heat shock 70 kDa protein cognate 4-like* (LOC109598691), *cytochrome P450 4c3-like* (LOC109598257), and *plasminogen receptor* (LOC109597380) (Figure 6). The gene LOC109601627 was not significantly differentially expressed among the four groups, and the gene LOC109605651 showed a different expression trend in the four groups compared to the results of RNA-Seq (Figure 6). Notably, the expression of *HSP* genes including *heat shock 70 kDa protein cognate 5-like* and *heat shock 70 kDa protein cognate 4-like* was more significantly different in the 46 °C vs. 25 °C and 42 °C vs. 25 °C comparisons compared to the RNA-Seq results (Figure 6).

## 4. Discussion

The small hive beetle is originally native to the deserts of southern Africa and has now spread globally [11]. This study clearly indicated that *A. tumida* possesses a remarkable capacity to endure elevated temperatures. At 38 °C and 42 °C, the beetles exhibited extended survival durations (Figure 1). Even at higher temperature (46 °C) individuals survived for nearly one hour (Figure 1). The beetles were exposed to heat stress without access to food or water. Remarkably, they still exhibited high survival rates under these restrictive conditions, suggesting a stronger intrinsic tolerance to heat stress that may reflect their adaptability in natural environments. Another beetle species, *Monochamus alternatus* Hope, also showed a similarly high heat resistance [25]. Therefore, these findings suggest that *A. tumida*, like *Monochamus alternatus*, possesses an inherently high heat stress tolerance, which may represent a common adaptive trait among certain beetle species. This thermal resilience likely contributes to their survival and ecological success under fluctuating and extreme environmental conditions.

Transcriptome profiling revealed widespread transcriptional remodeling in *A. tumida* in response to rising temperatures. A total of 547, 1127, and 866 DEGs were identified at 38 °C, 42 °C, and 46 °C, respectively, in comparison to the control condition at 25 °C (Figure 3). This temperature-dependent increase in the DEG number up to 42 °C, followed by a slight decrease at 46 °C, and the expression of most heat shock genes showed a similar pattern characterized by an initial upregulation followed by downregulation (Figure 6). This pattern likely reflects the beetle’s physiological threshold for a transcriptional response before cellular damage becomes irreversible. Among the DEGs, 16, 25, and 5 *HSP* genes were differentially expressed at 38 °C, 42 °C, and 46 °C, respectively (Figure 5). Only five *HSP* genes were upregulated at 46 °C, indicating that extreme heat may inhibit the induction of protective molecular chaperones or cause transcriptional exhaustion. This aligns with previous reports in *Monochamus alternatus*, where the expression of the MaltHSP70-2 protein peaked at 40 °C but declined when the temperature exceeded the cellular tolerance thresholds [25]. Therefore, these findings suggest that *A. tumida* may exhibit transcriptomic plasticity in response to heat stress up to sublethal temperatures, beyond which the transcriptional machinery could become impaired. Additionally, the GO enrichment results revealed distinct biological responses across temperature gradients, which also supports this hypothesis. At 38 °C, the DEGs were mainly enriched in metabolic regulation and stress response genes (Figure S2A), implying an early-stage adaptation to moderate thermal conditions. At 42 °C, additional enrichment appeared in behavior, detoxification, antioxidant defense, and rhythmic processes genes, reflecting enhanced protective responses (Figure S2B). At 46 °C, stronger enrichment in immune response, cell junction, oxidative stress, and electron transport genes suggested intensified cellular and systemic responses to severe heat stress (Figure S2C).

*HSP* family genes have been widely recognized as key drivers of heat resistance in insect species and act as molecular chaperones that help maintain cellular homeostasis by stabilizing nascent polypeptides, in refolding misfolded proteins, and in preventing the aggregation of denatured proteins under elevated temperatures [26,27]. In particular, the *HSP70* genes have been widely implicated in cytoprotection against heat stress, and their expression has been reported to correlate with the survival rates in various insect taxa [25,28].

In the present study, 22 *HSP* genes, mainly the *HSP70*, *HSP90*, and *HSP20* family genes, were significantly upregulated after the heat stress treatments (Figure 5). One *HSP70* gene (Loc109602670) was the only DEG consistently upregulated across all temperature treatments. These findings, consistent with previous studies in other insect species [27], demonstrated that the *HSP* family genes play an important role in the heat stress response in insects. Notably, this supports the designation of the *HSP70* gene Loc109602670 as a “core responder” to heat stress and its potential as a molecular biomarker for thermal tolerance in insects.

Beyond *HSPs*, one of the most striking observations was the consistent enrichment in lysosome-related pathway genes among the DEGs under all heat stress conditions. This pathway, involved in cellular degradation, recycling of macromolecules, and stress adaptation, likely plays a complementary role in maintaining cellular integrity under thermal insult [29,30]. In the present study, dozens of DEGs involved in lysosome pathways were identified, and this key pathway was the top one pathway in all three comparisons, and its proportion increased with the temperature (Figure 4 and Figure 5). Notably, key genes in this lysosome pathway such as *Cathepsin L1-like* and *Lipase 3-like* were upregulated in the heat-stressed groups (Figure 5C). The *Cathepsin L1* family genes may be able to enhance proteolytic degradation during heat stress, thereby contributing to cellular proteostasis and survival under elevated temperatures [31]. *Lipase 3* is involved in lipid mobilization and energy homeostasis, supporting stress adaptation by facilitating the energy supply during thermal challenges [32]. Previous studies have reported that the *Lipase 3* gene is also involved in starvation stress in insects [33]. In the present study, it appears that *Lipase 3* may be simultaneously engaged in both starvation and heat stress responses. The processes in which it participates, namely, lipid mobilization and energy homeostasis, are likely to play important roles in protecting the small hive beetle under both stress conditions. Therefore, these findings suggest that the lysosome pathway in *A. tumida* could enhance the proteolytic activity and facilitate the removal of damaged proteins and organelles, thus contributing to cellular homeostasis.

Moreover, lysosomes are central components of the autophagy pathway, which is a key cellular process for recycling damaged proteins and organelles during stress adaptation [34]. Autophagy has been recognized as an integral part of the heat stress response in both mammals and insects, contributing to proteostasis and survival under elevated temperatures [30,35]. The consistent enrichment in lysosome-related genes observed in our study may also indicate the activation of autophagic processes in *A. tumida* during heat stress. While we did not directly assess autophagy-specific markers, these transcriptomic signatures suggest that autophagy, in coordination with HSP-mediated protein refolding, could represent an additional protective mechanism underlying the beetle’s thermotolerance. Future studies incorporating autophagy assays will be valuable for confirming this hypothesis. Additionally, starvation can also activate lysosomal pathways associated with autophagy [36], as well as the expression of *HSP* genes such as *HSP90* [37]. In this study, we sought to minimize the potential starvation-related impacts on gene expression; however, the possible influence of starvation on the expression of lysosomal pathway genes should still be taken into consideration.

The simultaneous upregulation of both *HSPs* and lysosomal proteases points toward a coordinated response involving both protein repair and degradation mechanisms. For example, *HSPs* coordinate their activity with that of antioxidant enzymes to confer thermal tolerance in *Pardosa pseudoannulata* [38]. In the present study, *HSPs* may also coordinate their activity with that of lysosome pathway genes and antioxidant enzymes during heat resistance. While *HSPs* act to refold or stabilize misfolded proteins, lysosomes may degrade irreparably damaged cellular components, collectively ensuring cellular survival under prolonged heat exposure. This dual response underscores the complexity of thermotolerance strategies in *A. tumida*, suggesting that successful adaptation involves a dynamic balance between cytoprotection and selective degradation. From an applied perspective, understanding the molecular basis of thermal adaptation in invasive pests is critical for developing predictive models of species distribution under climate change. Given the increasing frequency and intensity of heatwaves, species like *A. tumida* may gain competitive advantages over native species, leading to shifts in ecosystem dynamics and agricultural impacts. Consequently, molecular markers such as the *HSPs* and lysosomal genes identified in this study may serve as early warning indicators or targets for pest control strategies.

## 5. Conclusions

In conclusion, our study highlights the remarkable thermotolerance of *A. tumida* and provides novel insights into the molecular mechanisms underlying this trait. Through integrative analyses of survival data and transcriptomic profiles, we identified heat shock proteins and lysosome-associated genes as likely important contributors in the beetle’s adaptive response to high temperatures. These findings not only enhance our understanding of insect heat resistance but also inform strategies for managing invasive pests in a rapidly warming world.

## Data Availability

The raw data of RNA-Seq were uploaded to the NCBI database under the accession number NCBI Bioproject PRJNA1263418.

## Supplementary Materials

The following supporting information can be downloaded at: https://www.mdpi.com/article/10.3390/insects16080868/s1, Figure S1: Pearson correlation coefficient matrix of 12 RNA-Seq samples; Figure S2: GO classification of DEGs from heat-stressed and control groups; Table S1: Primers of 8 target genes and reference genes for qPCR; Table S2: Summary of RNA-Seq results for all samples; Table S3: DEGs of the 25 °C vs. 38 °C comparison; Table S4: DEGs of the 25 °C vs. 42 °C comparison; Table S5: DEGs of the 25 °C vs. 46 °C comparison.

## Abbreviations

The following abbreviations are used in this manuscript:

DEG	Differentially Expressed Gene
HSP	Heat Shock Protein
FPKM	Fragments per Kilobase of Exon per million Mapped Fragments
Log2FC	log2 Fold Change
GO	Gene Ontology
KEGG	Kyoto Encyclopedia of Genes and Genomes
