# Supplementary material for: Transcriptomic Plasticity in the Small Hive Beetle (Aethina tumida) Under Heat Stress
Source: Insects. 2025 Aug 21;16(8):868. doi: 10.3390/insects16080868 (PMC12386877; doi:10.3390/insects16080868)
Supplement: Supplementary file 1 [file insects-16-00868-s001.zip › Table S1.pdf]

**Table S1** Primers of 8 target genes and reference genes for qPCR

| Gene ID | Forward primer | Forward primer |
|---|---|---|
| *β*-actin | TCACCCACACTGTGCCCATCTACGA | CAGCGGAACCGCTCATTGCCAATGG |
| LOC109601627 | ATGTGCCTTACTTGGGTGATTTGG | GCAGTTGTCCTATTTGTATGACTTTGG |
| LOC109605651 | TGCCACAGGAGTACGACATAGC | TCCACGTTCATCTCAACTTCATCATC |
| LOC109602689 | GGTGTTGCTGGTTGGTATG | CTGCTAGGTTGCTTGCCGAAG |
| LOC109601676 | GGCTCATTACAAAGGCGATTTCAC | GAACCTACCTTCACACCATCCATC |
| LOC109600819 | GTGCTCGCCTGTCTCATGTTG | AAGGTTGAACTGCTTCTTCGGATC |
| LOC109598691 | GAGCGTGCTATGACCAAGGATAAC | TCAAGATACCGTTGGCATCAATGTC |
| LOC109598257 | ATTCCGCTAGTGAAGACACAGTAAC | AGTTTGGGCAAATCGTCTAGTTCC |
| LOC109597380 | ATCAGATGAGGGAGAGGGAAGTTG | AAAGTGCCTTAATGACGTTACCC |
