# Supplementary material for: Transcriptomic Plasticity in the Small Hive Beetle (Aethina tumida) Under Heat Stress
Source: Insects. 2025 Aug 21;16(8):868. doi: 10.3390/insects16080868 (PMC12386877; doi:10.3390/insects16080868)
Supplement: Supplementary file 1 [file insects-16-00868-s001.zip › Table S2.pdf]

Table S2 Summary of RNA-Seq in all samples

Samples	Clean reads	Clean bases	GC Content	%≥Q30
T25-R1	21,122,274	6,311,186,084	44.13%	95.27%
T25-R2	19,545,095	5,841,897,532	44.46%	95.36%
T25-R3	21,078,468	6,292,970,012	44.25%	95.61%
T38-R1	19,943,707	5,958,932,712	44.08%	95.39%
T38-R2	21,488,492	6,417,168,962	43.85%	95.63%
T38-R3	19,879,854	5,942,841,930	43.73%	95.04%
T42-R1	21,283,462	6,358,859,658	44.39%	95.33%
T42-R2	24,797,578	7,409,759,020	44.79%	95.14%
T42-R3	20,457,293	6,111,306,114	44.48%	95.36%
T46-R1	21,170,120	6,327,978,380	44.14%	92.54%
T46-R2	22,827,849	6,827,477,064	44.08%	92.40%
T46-R3	21,760,015	6,502,042,914	44.38%	93.12%
