# Supplementary figures and images for: Transcriptomic Plasticity in the Small Hive Beetle (Aethina tumida) Under Heat Stress
Source: Insects. 2025 Aug 21;16(8):868. doi: 10.3390/insects16080868 (PMC12386877; doi:10.3390/insects16080868)

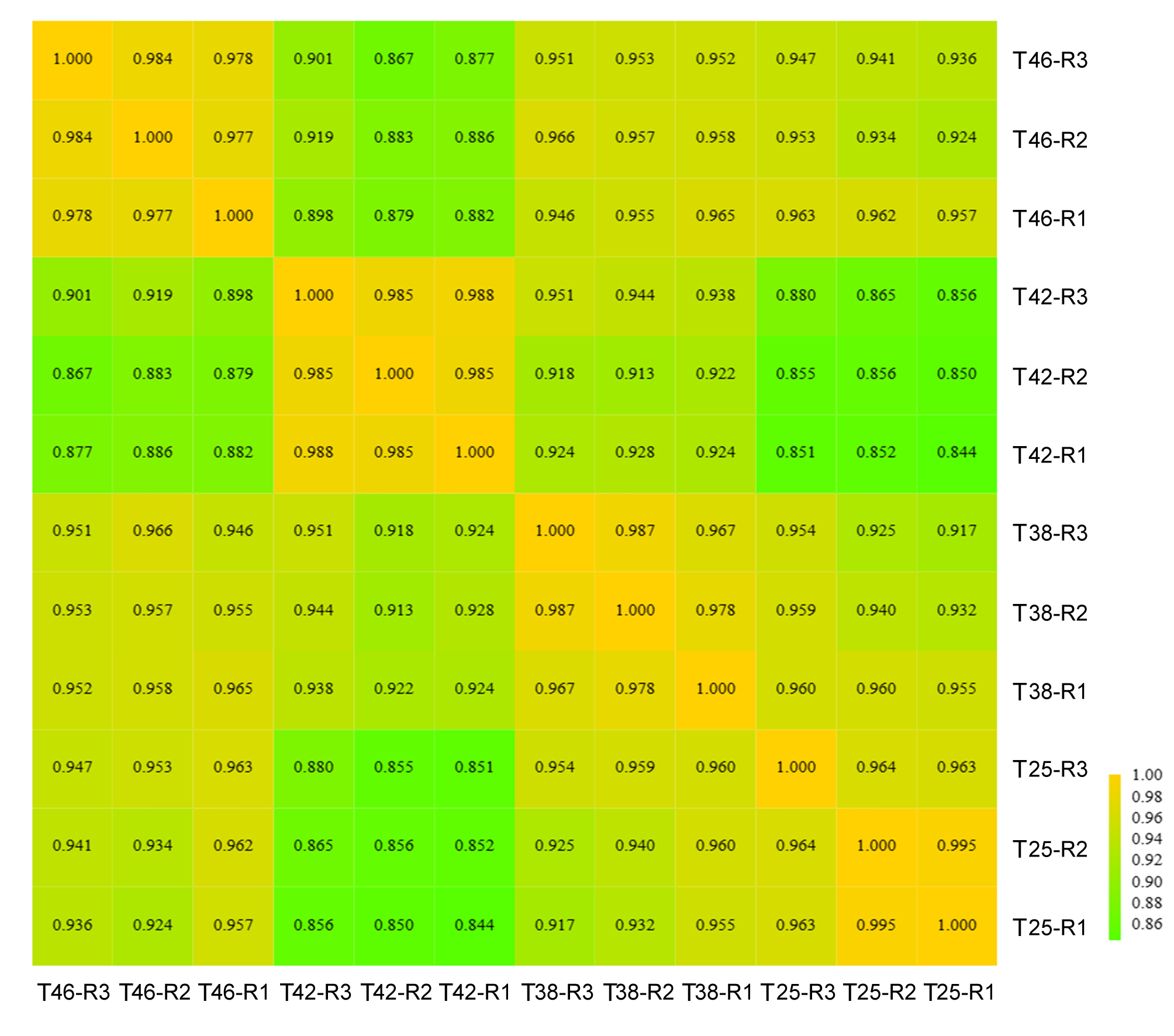

Supplement: Supplementary file 1 [file insects-16-00868-s001.zip › Figure S1.tif]

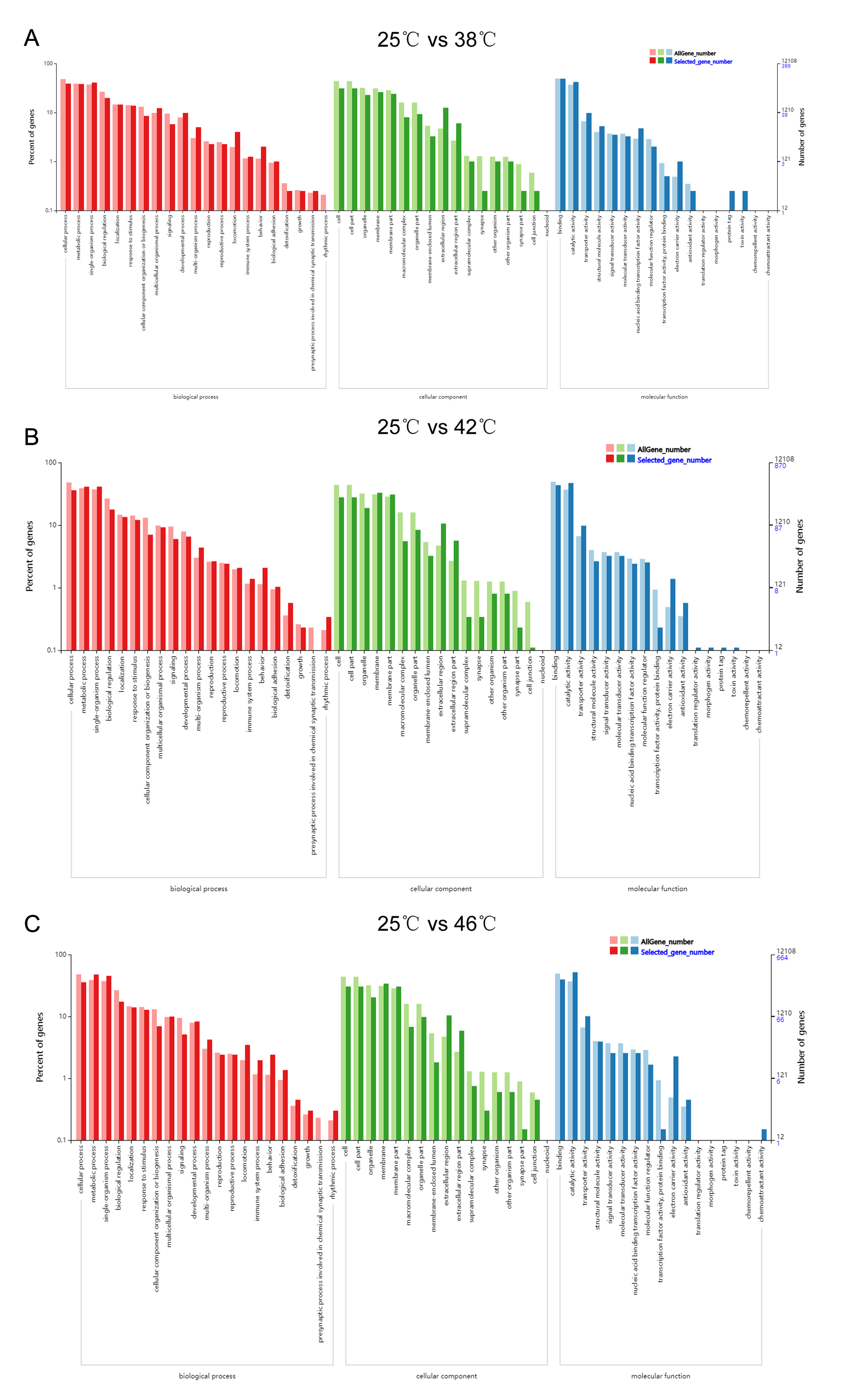

Supplement: Supplementary file 1 [file insects-16-00868-s001.zip › Figure S2.tif]
